# The values of systemic immune-inflammation index and neutrophil-lymphocyte ratio in predicting testicular germ cell tumors: A retrospective clinical study

**DOI:** 10.3389/fonc.2022.893877

**Published:** 2022-09-16

**Authors:** Shuo Wang, Xiao Yang, Ziyi Yu, Peng Du, Yudong Cao, Yongpeng Ji, Jinchao Ma, Yong Yang

**Affiliations:** Key Laboratory of Carcinogenesis and Translational Research (Ministry of Education), Urological Department, Peking University Cancer Hospital and Institute, Beijing, China

**Keywords:** germ cell tumors, neutrophil/lymphocyte ratio, systemic immune-inflammation index, red cell distribution, inflammation

## Abstract

**Purpose:**

To determine whether complete blood count (CBC) based inflammatory parameters can be used as markers predicting testicular germ cell tumors (TGCT).

**Material and methods:**

Between 2013 to 2018 the data of 58 patients with testicular TGCT undergoing radical orchiectomy and 54 malignancy-free healthy men were retrospectively analyzed as tumor group and control group. Patient baseline characteristics including age, pathological stage and pre-surgery CBC based inflammatory parameters including neutrophil/lymphocyte ratio (NLR), platelet/lymphocyte ratio (PLR), lymphocyte/monocyte ratio (LMR), systemic immune-inflammation index (SII), lymphocyte ratio (LR), neutrophil ratio (NR), mean platelet volume (MPV) and red cell distribution width (RDW) were analyzed and compared between tumor group and control group. Receiver operating characteristic (ROC) curve were used analyzing data with significantly difference to assess the discriminative ability of the markers for TGCT, area under the curve (AUC), cut-off value, sensitivity and specificity were calculated. The binary logistic regression model was used to evaluate the association between significant inflammatory markers and risk of TGCT.

**Results:**

Mean age of the tumor and control group was 41.1 ± 15.36 and 44.89 ± 9.2 years, respectively. Mean NLR, SII and RDW were significantly higher in tumor group compared with control group with P=0.005, P=0.001 and P=0.016, respectively; there were no significantly differences of age, PLR, LMR, LR, NR, MPV and RDW between groups. The ROC curve for NLR, SII and RDW was plotted in the diagnosis of TGCT and tumor progression, the cut-off value for NLR, SII and RDW were found as 3.38 (AUC: 0.704, sensitivity=51.4%, specificity=88.6%, P=0.003), 881.24 (AUC: 0.725, sensitivity=45.7%, specificity=91.4%, P=0.001) and 0.14 (AUC: 0.63, sensitivity=28.6%, specificity=97%, P=0.063), respectively. Patients were divided into two groups according to the threshold values, respectively. By using the multivariable logistic regression models, NLR ≥ 3.38 (OR, 5.86; 95% CI, 1.67-20.65, P=0.006) and SII ≥ 881.24 (OR, 4.89; 95% CI, 1.48-15.32, P=0.009) were independent risk factors predicting TGCT. Significantly statistical difference of pathological stage was also found between groups with respect to NLR cut-off values (P=0.034) and SII cut-off values (P=0.049). Combined the data together, NLR and SII both exhibited good differential diagnosis potential which could be used as markers predicting the TGCT.

**Conclusion:**

As the CBC based inflammation parameters, both NLR and SII could be used as effective tumor markers predicting the TGCT, and higher NLR and SII are associated with higher pathological stage. In addition, SII is a more powerful tool among these two inflammatory markers.

## Introduction

Testicular tumor is quiet a rare disease, 90-95% of testicular tumors are testicular germ cell tumors (TGCT) (1). The main methods for diagnosis of testicular tumors are physical examination, radiography, ultrasound and biochemical tumors markers including alpha-fetoprotein (AFP), human chorionic gonadotropin (HCG) and lactate dehydrogenase (LDH) (2). However, these markers are not very specific, AFP and HCG are increased in only 40-60% patients with TGCT, while HCG elevation can be detected in only 30% of seminoma (3), therefore, false positive and negative result are often observed by those examinations, so other simple, inexpensive, easily applicable and more accurate markers are needed in the clinical approach.

Inflammatory markers are with low cost and can be easily available from the routine hemorrhagic data, and the relationship between inflammation and various tumor is confirmed by several studies, it may play an essential role in regulating the progression of the cancer by stimulating or suppressing tumor cells (4, 5). When regrading to urological tumors, several studies demonstrated that inflammatory factors may be associated with progression and prognostic of renal cancer, bladder cancer and prostate cancer (6). Especially for neutrophil to lymphocyte (NLR), as one of the most important inflammatory markers, it has been reported to be closely related with recurrence and prognosis of kidney and bladder cancer (6, 7). However, few studies have detected the association of inflammation with testicular tumor, and among the published papers the conclusions are still controversial, further studies are needed to detect whether there is an association between inflammation and testicular tumors, and whether inflammatory parameters can be used as predicting markers for testicular TGCT. The aim of this study is to clarify whether complete blood count (CBC) based inflammatory markers including lymphocyte ratio (LR), platelet/lymphocyte ratio (PLR), lymphocyte/monocyte ratio (LMR), systemic immune-inflammation index (SII), lymphocyte ratio (LR), neutrophil ratio (NR), mean platelet volume (MPV) and red cell distribution width (RDW) could be used as serum markers for predicting TGCT, if so, great clinical values will be provided in predicting testis TGCT for patients with testis masses before surgery.

## Materials and methods

### Methods

The data of 58 patients who underwent inguinal orchiectomy as tumor group and 54 malignancy-free healthy men who underwent physical examination as control group in Beijing Cancer Hospital between 2013 and 2018 were analyzed retrospectively. The studies involving human participants were reviewed and approved by Institutional Review Board of Peking University Cancer Hospital & Institution in April 2020 (protocol code 2018KT27). Pathological confirmed testicular TGCT of stage I-III and malignancy-free healthy men were included in this study and defined as tumor and control group. Patients with acute infections, chronic inflammation disease, malignancies beside testicular GTCs, hematological disorders and blood product administration recently were excluded.

Blood samples of the patients were taken within the pre-surgery 24h. Hematological parameters including LR, NR, MPV and RDW were evaluated with peripheral blood samples, and NLR, PLR, LMR and SII were calculated by using the numbers of blood cell counts based systemic markers of inflammation. SII (SII= platelet × neutrophil/lymphocyte) has been presented as a combination of NLR and PLR and shown to suggest oncological results for many tumors (8, 9). The age of all patients and clinicopathological data including tumor stage I-III and histopathology according to current testicular tumor guidelines (2019 TNM classification) (10) were recorded. Stage I tumors localized to the testis, stage II tumors were with positive localized lymph nodes, stage III tumors were with distant metastasis, and differences between inflammatory markers were assessed and calculated.

### Statistical analysis

Measurement data conforming to normal distribution analyzed by Shapiro-Wilk test are represented as Mean ± SD, independent sample t test and Box-plot graphics are used for comparison between groups. Data on categorical variables are presented as frequency with percentages and differences among groups are analyzed with Pearson’s chi-square test or Fisher exact test as appropriate. Receiver operating characteristic (ROC) curve analyses were performed to assess the discriminative ability of the inflammatory markers for TGCT. The cut-off points for markers were defined by a criterion based on Youden’s index defined as YI(c)=max c [Se(c)+Sp(c)-1] and corresponding specificity- sensitivity levels were provided. The binary logistic regression model (univariable and multivariable analysis) was used to evaluate the association between significant factors and risk of TGCT, which were all compared with reference group (Ref). The software used to run the analysis was IBM-SPSS version 20. All tests were two-sided, P<0.05 was considered to be the threshold for statistically meaningful differences.

## Results

### Clinicopathological characteristics of patients

A total of 112 patients were included in this study after determine the inclusion and exclusion criteria with ages ranging from 20 to 73 years. The mean age was 39.9 ± 13.23 years. The cases were divided into two groups: tumor (n=58) and control (n=54). Mean age of tumor and control group was 41.1 ± 15.36 and 44.89 ± 9.2 years, respectively. Demographic, clinicopathologic and pathological stage features of patients with TGCT were summarized in **Table 1**. For metastatic cases, the international germ cell tumor cancer collaborative group (IGCCCG) has identified three prognostic groups: good, intermediate and poor risk (11). Among 9 patients with stage II, according to IGCCCG, 7 (77.8%) were with good prognostic (6 seminoma, 1 non-seminoma), 2 (22.2%) were with intermediate prognostic (2 non-seminoma); among 21 patients with stage III, 12 (57.1%) were with good prognostic (5 seminoma, 7 non-seminoma), 5 (23.8%) were with intermediate prognostic (2 seminoma, 3 non-seminoma), 4 (19.0%)were with poor prognostic (4 non-seminoma).

**Table 1:** Demographic and clinicopathologic features of patients with testis tumor.

	Number	Seminoma	Non-seminoma		
			Immature Teratoma	Choriocarcinoma	Mix germ cell
Stage, n (%)	58	35	9	3	11
I	28 (48.3)	22 (62.9)	3 (33.3)	0 (0)	3 (27.3)
II	9 (15.5)	6 (17.1)	0 (0)	1 (33.3)	2 (18.2)
III	21 (36.2)	7 (20)	6 (66.7)	2 (66.7)	6 (54.5)

### Analysis of clinical and CBC based parameters for predicting TGCT

**Figure 1:**
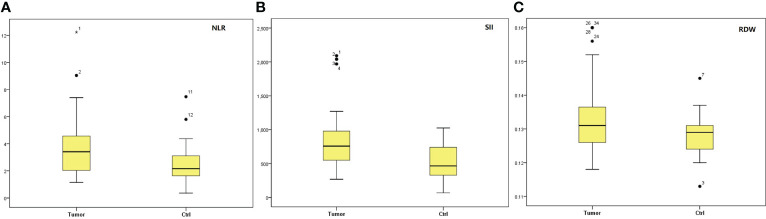
Box-blot graphics of markers of NLR, SII and RDW for testis tumors and ctrl group. **(A)** NLR, **(B)** SII, **(C)** RDW. NLR, neutrophil/lymphocyte ratio; SII, systemic immune-inflammation index; RDW, red cell distribution.

In tumor group, the median NLR, SII and RDW levels were significantly higher than those in control group (P=0.005; P=0.001; P=0.016) as shown in **Figure 1** and **Table 2**. There were no significantly statistical differences between groups in terms of ages, PLR, LMR, LR, NR and MPV as shown in **Table 2**.

**Table 2:** Descriptive statics and comparison of CBC based parameters with respect to groups.

Variables	Tumor group (n = 58)	Control group (n = 54)	P-value
Age (years)	41.1 ± 15.36	44.89 ± 9.2	0.209
NLR (%)	3.79 ± 2.36	2.45 ± 1.4	0.005
PLR (%)	173.87 ± 62.9	157.68 ± 54.26	0.339
LMR (%)	4.11 ± 2.17	5.1 ± 2.11	0.616
SII (%)	870.39 ± 496.35	526.55 ± 263.64	0.001
LR (%)	24.35 ± 8.06	25.7 ± 9.8	0.533
NR (%)	68.04 ± 9.4	64.8 ± 10.7	0.184
MPV (10^3^/μL)	9.86 ± 1.24	9.16 ± 1.82	0.062
RDW (%)	13.41 ± 1.2	12.83 ± 0.6	0.016

NLR, neutrophil/lymphocyte ratio; PLR, platelet/lymphocyte ratio; LMR, lymphocyte/monocyte ratio; SII, systemic immune-inflammation index; LR, lymphocyte ratio; NR, neutrophil ratio; MPV, mean platelet volume; RDW, red cell distribution; CBC, complete blood count.

**Figure 2:**
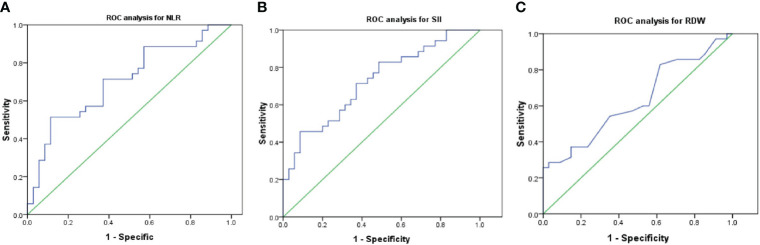
Optimal cut-off values and ROC analyses for NLR **(A)**, SII **(B)** and RDW **(C)**. NLR, neutrophil/lymphocyte ratio; SII, systemic immune-inflammation index; RDW, red cell distribution; ROC, receiver operating characteristic.

The ROC curve for NLR, SII, and RDW was plotted in the diagnosis of testicular tumor as shown in **Table 3** and **Figure 2**. AUC for NLR in tumor group was 0.704 which was significantly higher than 0.5 (P=0.003), with a threshold value of 3.38 and sensitivity 51.4% and specificity 88.6%; AUC for SII in tumor groups was 0.725 which was significantly higher than 0.5 (P=0.001), with a threshold value of 881.24 and sensitivity 45.7% and specificity 91.4%; AUC for RDW in tumor groups was 0.63 which was higher than 0.5 (P=0.063), with a threshold value of 0.14 and sensitivity 28.6% and specificity 97%, together NLR and SII exhibited good differential diagnosis potential which could be used as adjuvant tool in the prediction of testicular germ cell tumors.

**Table 3:** Cut-off, AUC, sensitivity and specificity values of NLR, SII and RDW.

Variables	AUC	Cut-off	Sensitivity	Specificity	95% CI	P-value
Preop NLR	0.704	≥ 3.38	51.4%	88.6%	0.581-0.826	0.003
Preop SII	0.725	≥ 881.24	45.7%	91.4%	0.608-0.842	0.001
Preop RDW	0.63	≥ 0.14	28.6%	97%	0.499-0.762	0.063

AUC, Area under the curve; NLR, neutrophil/lymphocyte ratio; SII, systemic immune-inflammation index; RDW, red cell distribution.

Then patients were divided into 2 groups according to the threshold value of NLR and SII, univariable and multivariable logistic regression models were used to evaluate the association between factors and risk of TGCT. In univariable analysis, NLR ≥ 3.38 (OR, 4.5; 95% CI, 1.52-13.30, P=0.007) and SII ≥ 881.24 (OR, 5.33; 95% CI, 1.55-18.30, P=0.008) were risk factors predicting TGCT as shown in **Table 4**; In multivariable analysis, NLR ≥ 3.38 (OR, 5.86; 95% CI, 1.67-20.65, P=0.006) and SII ≥ 881.24 (OR, 4.89; 95% CI, 1.48-15.32, P=0.009) were independent risk factors predicting TGCT as shown in **Table 4**.

**Table 4:** Univariable and multivariable analyses for predicting testicular germ cell tumors.

	Univariable analysis			Multivariable analysis		
	Tumor vs. Control			Tumor vs. Control		
	OR	95% CI	P value	OR	95% CI	P-value
NLR						
<3.38	1 (Ref)	1 (Ref)		1 (Ref)	1 (Ref)	
≥3.38	4.5	1.52-13.30	0.007	5.86	1.67-20.65	0.006
SII						
<881.24	1 (Ref)	1 (Ref)		1 (Ref)	1 (Ref)	
≥881.24	5.33	1.55-18.30	0.008	4.89	1.48-15.32	0.009

Ref, reference; NLR, neutrophil-lymphocyte ratio; SII, systemic immune-inflammation index.

We also compared the CBC based parameters between seminomatous testicular germ cell tumors (sTGCT) and non-seminomatous testicular germ cell tumors (nsTGCT), but there seemed no significantly statistical differences of NLR, PLR, LMR, SII, LR, NR, MPV and RDW between these two groups, P=0.128, 0.258, 0.413, 0.085, 0.234, 0.194, 0.192 and 0.116, respectively, as shown in **Table 5**.

**Table 5:** Comparison of CBC based parameters between sTGCT and nsTGCT.

Variables	sTGCT (n = 35)	nsTGCT (n = 23)	P-value
NLR (%)	2.69 ± 1.36	2.95 ± 2.03	0.128
PLR (%)	165.97 ± 42.8	169.97 ± 53.16	0.258
LMR (%)	4.58 ± 1.88	4.99 ± 2.02	0.413
SII (%)	718.40 ± 289.25	629.65 ± 159.63	0.085
LR (%)	29.25 ± 7.85	24.37 ± 6.82	0.234
NR (%)	67.94 ± 8.13	65.18 ± 12.37	0.194
MPV (10^3^/μL)	9.34 ± 2.14	9.56 ± 1.92	0.192
RDW (%)	14.41 ± 1.31	11.92 ± 1.26	0.116

NLR, neutrophil/lymphocyte ratio; PLR, platelet/lymphocyte ratio; LMR, lymphocyte/monocyte ratio; SII, systemic immune-inflammation index; LR, lymphocyte ratio; NR, neutrophil ratio; MPV, mean platelet volume; RDW, red cell distribution; CBC, complete blood count; sTGCT, seminomatous testicular germ cell tumors; nsTGCT, non-seminomatous testicular germ cell tumors.

Among TGCT patients, post-operative CBCs bases parameters including SII and NLR (1 month after inguinal orchiectomy) were also collected, when compared with pre-operative SII and NLR, the level of post-operative SII significantly reduced compared with pre-operative SII (870.39 ± 496.35 vs 721.21 ± 328.17, P=0.032), but there was no difference of NLR between groups (3.79 ± 2.36 vs 3.23 ± 1.89, P=0.089).

### Distribution of pathological stage with regard to cut-off value of SII, NLR

Comparison of age and pathological stage between the patients with respect to NLR (< 3.38 and ≥3.38) or SII cut-off values (<881.24 and ≥881.24) are shown in **Table 6**. Significantly Statistical difference of pathological stage was found between groups with respect to NLR cut-off values (P=0.034) and SII cut-off values (P=0.049), when NLR ≥3.38 or SII ≥881.24 more patients are with stage II and stage III. There was no significantly difference of age between groups.

**Table 6:** Distribution of descriptive properties and comparison of Clinical parameters between the patients with respect to NLR <3.38 and ≥3.38, SII<881.24 and ≥881.24.

Variables	NLR<3.38 (n = 31)	NLR≥3.38 (n = 27)	P value	SII<881.24 (n = 34)	SII≥881.24 (n = 24)	P-value
Ages (years)	33.6 ± 9.8	45.1 ± 14.8	0.011	37.16 ± 14.17	42.25 ± 13.11	0.281
Stage, n (%)			0.034			0.049
IA, B, S	19 (61.3)	9 (33.3)		20 (58.8)	8 (33.3)	
II+III	12 (38.7)	18 (66.7)		14 (41.2)	16 (66.7)	

NLR, neutrophil/lymphocyte ratio; SII, systemic immune-inflammation index.

### Comparison of SII, NLR in this study with traditional biomarkers and microRNA reported by other studies in TGCT.

We compared the data of SII, NLR of our study with traditional biomarkers (AFP, HCG and LDH) and 3 most important kinds of miR related with TGCT (miR-371-3p, miR-372-3p and miR-373-3p) reported by other studies. SII and NLR were with comparable or better values of sensitivity compared with AFP, HCG and LDH, and are with comparable values of specific compared with miR cluster, but the sensitivity of SII, NLR is much lower than that of miR cluster. Markers including SII, AFP, HCG, LDH and miR cluster were all decreased after orchiectomy. The levels of these 3 kinds of markers could be used for predicting the stages of the tumors **(** **Table 7** **)**. Due to the methodological limitations of our study, some data were not available according to the recent methods, further studies were needed.

**Table 7:** Comparison of NLR, SII in present study with traditional biomarkers and miR markers reported by other studies.

Variables	NLR	SII	AFP, HCG, LDH	miR-371-3p	miR-372-3p	miR-373-3p
Predicting role in TGCT	Yes	Yes	Yes	Yes (12–16)	Yes (12–14, 16, 17)	Yes (12–14, 17)
Sensitivity (%)	51.4%	45.7%	AFP:13.6-47.1% (14–16) bHCG:9.3-64.7% (14–16) LDH:52.9% (16) AFP+bHCG+LDH: 50.4% (18);	70.8-88.7% (14, 15, 18)	82-87.5% (16, 17)	59% (17, 18)
Specificity (%)	88.6%	91.4%	NA	93.4-99% (15, 18)	65-94% (16, 17)	91% (17)
Markers’ level decreased after orchiectomy	No	Yes	Yes (14, 15) ^[1]^	Yes (14, 15)	Yes (14, 15)	Yes (14, 15)
Consistent in fluids and tissues	NA	NA	NA	Yes ^[2,3]^	Yes ^[2,3]^	Yes ^[2,3]^
Stages distinguish	Yes	Yes	Yes ^[1]^	Yes (15, 18)	NA	Yes (17)
Histological distinguish between sTGCT and nsTGCT	No	No	Yes ^[1]^	Yes (15)	NA	NA
Specific expressed in TGCT	No ^[4]^	No ^[5,6]^	No ^[1]^	Yes (12–16)	Yes (12–14, 16, 17)	Yes (12–14, 17)

NA, not applicable; TGCT, testicular germ cell tumors; sTGCT, seminomatous testicular germ cell tumors; nsTGCT, non-seminomatous testicular germ cell tumors.

^1^Paolo Pedrazzoli, Giovanni Rosti, Elenora Soresini, et al. Serum tumour markers in germ cell tumours: from diagnosis to cure. Crit Rev Oncol Hematol. 2021, 159:103224.

^2^Almstrup K, Lobo J, Morup N, et al. Application of miRNAs in the diagnosis and monitoring of testicular germ cell tumours. Nat Rev Urol. 2020;17:201-203.

^3^Lobo J, van Zogchek MJ, Nuru MG, et al. Combining hypermethylated RASSF1A detection using ddPCR with miR-371a-3p testing: an improvement panel of liquid biomarkers for testicular germ cell tumor patients. Cancer (Basel). 2021, 13:5228.

^4^Wang S, Ji Y, Chen Y, et al. The values of systemic immune-inflammation index and neutrophil-lymphocyte ratio in the localized prostate cancer and benign prostate hyperplasia: a retrospective clinical study. Front Oncol. 2022, 11:812319.

^5^Kao SC, Pavlakis N, Harvie R, et al. High blood neutrophil-to-lymphocyte ratio is an indicator of poor prognostic in malignant mesothelioma patients undergoing systemic therapy. Clin Cancer Res. 2010, 16:5805-5813.

^6^Viers BR, Boorjian SA, Frank I, et al. Pretreatment neutrophil-to-lymphocyte ratio is associated with advanced pathologic tumor stage and increased cancer-specific mortality among patients with urothelial carcinoma of the bladder undergoing radical cystectomy. Eur Urol. 2014, 66:1157-1164.

## Discussion

The relationship between inflammation and cancer has long been known, a range patterns of cellular immune response to different histological tumors types are reported. In 1863, Virchow hypothesized that chronic inflammation could irritate cell proliferation along with the inflammation leading to cancer occurs (19). The immunocompetent cells that infiltrate tumors are mostly T-lymphocytes and microphages, with a few B-lymphocytes and NK-cells (20). Neutrophils mediate inflammation through various biochemical mechanisms such as platelet aggravating factors and release of arachidonic acid metabolites, and lymphopenia is associated with cortisol induced stress response (21). Other systemic inflammatory markers including C-reactive protein, leukocyte and cytokines were reported to be independent prognostic factors for patients with malignant (22). In recently, in addition to those inflammatory markers some papers reported that several CBC based parameters including NLR, PLR and SII are associated with the formation and progression of several kinds of malignant tumor (22, 23). Inflammation caused neutrophil response increasing and lymphocyte suppression led to the high NLR supported the development of malignant tumor by inhibiting the antitumor immune response (24). In preclinical experiment, data shown that increased neutrophils could stimulate tumor growth through different mechanisms (25). PLR has also been proved to be effective markers of system inflammation, and PLR combined with NLR are thought to be reliable independent prognostic factors in patients with malignancy (26). SII is a new developed joined tool combined with neutrophils, lymphocytes and platelet, recently it is used to assess the information of progression and prognostic in patients with malignant tumor (8, 27). Compared with NLR and PLR, SII is thought to be a more powerful tool combining three independent prognostic factors in cancer (28). And high SII has been demonstrated to be associated with tumor progression and poor outcomes in various kinds of cancer (29).

Meanwhile, the presence and consequences of cancer-related systemic inflammatory response have been investigated in various urologic cancers. Studies assessed that inflammation might be associated with development of urinary system malignancies such as bladder cancer, renal cancer and prostate cancer (30). A meta-analysis evaluating the relationship between NLR and prognostic found that high NLR was associated with poor prognosis in urological tumors beyond testis tumor (31). Bell et al. investigated the nature of inflammatory cell infiltrated in 10 testicular seminomas, found all of the 10 tumors contained a slight to marked inflammatory cell infiltrate at the periphery of the tumor, indicated that 2 types of immune-inflammatory reactions may play crucial role in the testicular seminomas (32), as sensitive markers reflecting inflammatory-immune status of the body NLR and SII may be affected, but the number of studies on the association of inflammation with testicular tumors is really small, most evaluations were performed using NLR and PLR, and the results are controversial. A study comparing patients with early-stage testicular tumors with healthy men confirmed that NLR above 2.7 should be considered the diagnosis of tumor (33). Another study demonstrated pre-operative CBC based parameters including NRL, RDW, MPV, LR and NR are all associated with progression and prognostic in patients with testicular tumors (34). Some other literatures demonstrated that TGCT were associated with prominent lymphocytic infiltrate (35, 36). In contrast, in some other studies, the conclusion was different. In a study assessing the association between inflammation factors and progression and prognostic in patients with TGCT, no correlation was found between NLR and stage, cancer specific survival (CSS) time and progression free survival (PFS) time (37). As a powerful tool in predicting various cancers, few studies reported the relationship between SII and testicular tumors. Only in recently years, a retrospective study demonstrated that high SII (≥1003) was associated with poor outcomes in patients with TGCT (23). Therefore, it is still controversial to define whether inflammation factors are associated with occurrence and progression of testicular tumors and which parameters can be considered as the most effective predictors for diagnosis of testicular tumors and disease progression, and further studies are needed detecting the role of SII in TGCT.

The most usually used methods for diagnosis of testicular germ cell tumors included testicular ultrasound, serum AFP and HCG, but false positive and negative result are often observed by these examinations, it is difficult for ultrasound to separate germ cell tumors from other testicular tumors, and the level of AFP and HCG are easily influenced by other diseases, eg: hepatitis, hepatoma tumors, ovarian tumors, stomach tumors (38, 39), so other simple, inexpensive and easily applicable markers are needed in the clinical approach. New miR based serum biomarkers including miRNA-367-3p, 371a-3p, 372-3p and 373-3p have shown great potential with high sensitivity and specificity for predicting TGCT, compared with miR cluster, the specific of SII and NLR are comparable, but the sensitivity is much lower (12–18, 40), inflammatory markers in this study seemed have no too much advantage compared with miR cluster, but the extraction of miR from the fluid needs specific equipment and professional researchers, not all the centers could perform that, for SII and NLR, they could be calculated based on CBC, easily available and much cheaper compared with extraction of miR cluster. Therefore, SII and NLR could be considered as valuable markers, and they were more likely becoming the common clinical method for predicting TGCT compared with miR cluster. In this study, we sought to detect the potential association between pre-operative CBC based blood count parameters including NLR, PLR, LMR, SII, LR, NR, MPV and RDW with TGCT. After analyzing the data, the major findings of our study are: 1) NLR, SII and RDW are significantly higher in patients with TGCT compared to tumor-free healthy patients; 2) NLR and SII all could be used as effective bio-markers for the prediction of TGCT, while SII seemed to be a more favorable choice due it got the largest AUC area of 0.725 compared with NLR of 0.704; 3) More than half of the cases in this study are with stage II or III, due to the small sample size, we did not compare the data of tumor group with control group according to the pathological stage, separately. However, after grouping the patients by cut-off value of SII or NLR, we found that NLR above 3.38 or SII above 881.24 are associated with higher pathological stage, the volume of the disease may affect the inflammatory parameters. The outcome of NLR including its role in predicting testicular tumor and cut-off value are mostly conformal with data reported by literatures (33, 41). While few studies have detected the correlation between SII and TGCT, in our study, for the first time in the literature, the role of SII in predicting TGCT was analyzed and the results indicated that SII had diagnostic value in detection TGCT, which was really help for patients with testis mass who refused performing surgery or biopsy, and the cut-of value of SII was similar with that reported by Michal et al. (23).However, according to our study, inflammatory markers seemed not able to distinguish the pathological types of the TGCT, for there were no significant differences of CBC based parameters between sTGCT and nsTGCT.

Meanwhile, our study has some limitations. As a retrospective study, the sample size is relatively small due to the low incidence of testicular tumor, only 58 cases with GTCs are included. And our study lacks the following-up data while most of the patients included are seminoma making it very difficult to measure the factors associated with prognostic. And one single time point was used for measuring the biomarkers leading to the inaccurate of the data collected, it can be strengthened by collecting blood samples at different pre-operative sets. Extended sample size and following-up data were needed in further research.

## Conclusions

In conclusion, this study demonstrated that NLR and SII were all effective markers for urologists predicting the occurrence of TGCT, as they showed superior performance in detecting TGCT; in addition, SII is a more powerful tool among these 2 inflammatory factors for predicting TGCT. Extended sample size and prospective studies are needed.

## Data availability statement

The raw data supporting the conclusions of this article will be made available by the authors, without undue reservation.

## Ethics statement

The studies involving human participants were reviewed and approved by Institutional Review Board of Peking University Cancer Hospital & Institution in April 2020 (protocol code 2018KT27). Written informed consent for participation was not required for this study in accordance with the national legislation and the institutional requirements.

## Author contributions

DP and WS designed the study. SW, XY, and ZY made the same contribution in this study. SW, XY, ZY, YJ, YC, JM and YY performed the study and analyzed the data. PD, SW, XY and ZY wrote the manuscript draft and revised the manuscript. All authors contributed to the article and approved the submitted version.

## Funding

Beijing Municipal Science & Technology Commission. No. Z181107001718142. Science Foundation of Peking University Cancer Hospital. No.2021-7; Wu Jie Ping Medical Foundation. No. 320.6750.2020-19-2.

## Conflict of interest

The authors declare that the research was conducted in the absence of any commercial or financial relationships that could be construed as a potential conflict of interest.

## Publisher’s note

All claims expressed in this article are solely those of the authors and do not necessarily represent those of their affiliated organizations, or those of the publisher, the editors and the reviewers. Any product that may be evaluated in this article, or claim that may be made by its manufacturer, is not guaranteed or endorsed by the publisher.
